# Review of the type series of *Pterocles exustus* Temminck, 1825 (Aves, Pterocliformes, Pteroclidae) and designation of a lectotype

**DOI:** 10.3897/zookeys.580.7892

**Published:** 2016-04-12

**Authors:** Christophe Gouraud, Sylke Frahnert, Anita Gamauf, Steven van der Mije

**Affiliations:** 1 Unaffiliated, Franz Baumannweg 22, Top 39, A-6020 Innsbruck, Austria; 2 Museum für Naturkunde, Leibniz Institute for Evolution and Biodiversity Science, Invalidenstrasse 43, D-10115, Berlin, Germany; 3 Naturhistorisches Museum Wien, 1st Zoological Department – Ornithology, Burgring 7, A-1010 Vienna, Austria; 4 Naturalis Biodiversity Center, Postbus 9517, NL-2300 RA Leiden, The Netherlands

**Keywords:** Chestnut-bellied Sandgrouse, lectotypification, type locality

## Abstract

The type locality of *Pterocles exustus* Temminck, 1825, is ‘West coast of Africa, Egypt and Nubia’. This is problematic because it includes the type locality of *Pterocles exustus floweri* (Nicoll, 1921), which is Fayum, Egypt. In the interest of clarification and to preserve stability of nomenclature, a non-Egyptian specimen from the type series of *Pterocles exustus* is designated as lectotype of the taxon, and the type locality is restricted to Senegal.

## Introduction

The Chestnut-bellied Sandgrouse (*Pterocles exustus*) is a sedentary and nomadic species that naturally inhabits bare semi-deserts from Senegambia and Mauritania to Egypt, Sudan, Ethiopia and Somalia, as well as in Kenya, Tanzania, Arabian Peninsula and further east to Iran, Pakistan and India (del Hoyo and Collar 2014: 216). Six subspecies are currently recognized, with ranges as follows (Dickinson and Remsen Jr 2013: 81, del Hoyo and Collar 2014: 216):

- *Pterocles exustus exustus* Temminck in Temminck and Laugier de Chartrouse, 1825. Senegal, Gambia and Mauritania to Sudan,
- *Pterocles exustus floweri* Nicoll, 1921. Endemic to the Nile Valley (Egypt). This subspecies was believed to be extinct until its recent rediscovery (Khil et al. 2012),
- *Pterocles exustus ellioti* Bogdanov, 1881. SE Sudan E to Eritrea, N Ethiopia, Somalia. Includes *somalicus* Hartert, 1900,
- *Pterocles exustus olivascens* (Hartert 1909). SE South Sudan, SW Ethiopia, Kenya and N Tanzania. Includes *emini* (Reichenow in Heinroth 1919),
- *Pterocles exustus erlangeri* (Neumann 1909). SW Saudi Arabia, Yemen and Oman,
- *Pterocles exustus hindustan* R. Meinertzhagen, 1923. SE Iran, Pakistan and India. The name *hindustan* was introduced by Meinertzhagen to replace *Pterocles exustus orientalis* Hartert, 1900, pre-occupied by *Tetrao orientalis* Linnaeus, 1758. The type designated by Meinertzhagen has no type status as this author simply introduced a *nomen novum* (see Warren 1966: 129).

In his description, Temminck (in Temminck and Laugier de Chartrouse 1825: pl. 354 and 360 + text) wrote:

*The Sandgrouse described herein was sent in great number from West coast of Africa… Since then, Berlin and Frankfurt’s collections received specimens from travellers who explore Egypt for zoological discoveries purposes. Specimens received from this country by the Prussian naturalists* [Friedrich Wilhelm Hemprich (1796-1825) and Christian Gottfried Ehrenberg (1795-1876)] *and Mn. Rüppell* [Wilhelm Peter Eduard Simon Rüppell (1794-1884)] *do not differ from those received from Senegal* [translated from French].

The author ended with:

*Inhabits the West coast of Africa, Egypt and Nubia. Museums of Leiden, Paris, Berlin, Wien and Frankfurt* [translated from French].

All specimens used by Temminck to describe his *Pterocles exustus* constitute a type series and therefore all these specimens are syntypes (Art. 73.2 of the Code, see ICZN 1999). As far as we know, specimens comprising the type series can be found in the following institutions:

- Naturalis Biodiversity Center, Leiden (formerly RMNH, hereafter Naturalis): RMNH.AVES.87615 (adult male) and RMNH.AVES.87616 (adult female) from Senegal (van den Hoek Ostende et al. 1997: 82). These two specimens belong to the nominate subspecies.
- Naturhistorisches Museum, Wien (hereafter NMW): NMW 562 (male) and NMW 563 (female) from Senegal, received from Leiden in May 1821 (Schifter et al. 2007: 142). These two specimens belong to the nominate subspecies.
- Museum für Naturkunde , Leibniz Institute for Evolution and Biodiversity Science, Berlin (hereafter ZMB): ZMB 11416 (male) from Eritrea (Hemprich and Ehrenberg [April-July 1825]), ZMB 11417 (male) and ZMB 11418 (female) from Dongola (also spelled Dunqula), Sudan (Hemprich and Ehrenberg [January-June 1822]). Specimen ZMB 11416 belongs to *ellioti* whereas specimens ZMB 11417 and ZMB 11418 could belong either to *ellioti* or the nominal subspecies as both subspecies could overlap near Dongola.

Specimens of *Pterocles exustus* were sent by Hemprich and Ehrenberg to Berlin in their shipments numbered 7, 8 and 10 (Lichtenstein 1823c, 1824, 1826). The 7^th^ shipment arrived in March 1823 and comprised 22 adult specimens listed as *Pterocles senegalensis* M.H.C. Lichtenstein, 1823 (a name later on found to be pre-occupied, see below) in the first inventory list of the shipment (Lichtenstein 1823b) as well as in the unpublished catalogue 1857. The only adult specimens from this shipment found nowadays in Berlin are ZMB 11417 and ZMB 11418. 17 specimens, most likely never seen by Temminck, were given to an auction in 1823 comprising two specimens that ends up to Tartu museum, one to the anatomical collection in Berlin (at this time different than the zoological collection that became the ZMB, this specimen could not be retrieved) and two to Feliks Paweł Jarocki at the Zoology Cabinet of Warsaw University (Lichtenstein 1823b). The whereabouts of the remaining specimens from this auction are unknown as the whereabouts of the three last specimens of this shipment. The 8^th^ shipment that arrived in Berlin in May 1824 comprised only five specimens, also listed as *Pterocles senegalensis* M.H.C. Lichtenstein, 1823; they were never incorporated the Berlin’s collections and their whereabouts remain unknown. It is most likely that Temminck did not see these specimens. Finally, the 10^th^ shipment included a single specimen (ZMB 11416) and arrived in April 1826. It is impossible that this specimen formed part of Temminck’s type series because his planches coloriées 354 (male) and 360 (female) were issued with livraisons 60 (23 July 1825) and 61 (27 August 1825), respectively (Dickinson 2001: 47).

ZMB also houses two other specimens (ZMB 11572 and ZMB 11606) from Beni Suef, Egypt (Hemprich and Ehrenberg [September 1820-1825]). These specimens were catalogued separately as *Pterocles exustus* in the catalogue 1857 and are not part of the type series as they are young birds (“pullus” is mentioned in Berlin’s database) – Temminck described only the male and the female, but not the young.

There is another questionable specimen (ZMB 11419), a female collected (or traded) by “Verreaux” from Senegal. As it is not clear when it arrived in the collection, it cannot be decided whether it is a syntype or not. Having originated from Senegal, it belongs to the nominate subspecies.

- Musée George Sand et de la Vallée Noire, La Châtre (hereafter MLC): MLC.2011.0.1184 (female) from Upper Egypt (collected and given by Rüppell), designated as “Probable syntype” by Gouraud (2015). Under current taxonomy, this specimen belongs to *floweri*.

The Forschunginstitut und Naturmuseum Senckenberg (formerly Senckenberg Museum Frankfurt am Main, hereafter SMF) houses two specimens: SMF 23454 (male) and SMF 23455 (female) from Egypt and from the Rüppell Collection. There are no more data except that SMF 23455 bears the date “1843”. This is most likely a lapsus for 1834, as Rüppell was not in Africa in 1843. Indeed, the German explorer travelled four times in Africa: in 1817 (no collecting), in 1822-1827 and 1831-1834 (both trips providing important collections), and finally in 1849-1850 when only a handful of birds were collected (see Steinheimer 2005). SMF 23454 and 23455, most probably belonging to *floweri*, do not bear any original Rüppell’s label, corroborating that they are not part of the type series (Gerald Mayr in litt. 31 July 2015, Frank Steinheimer in litt. 6 August 2015).

Voisin et al. (2004) did not mention any type of this taxon in the Muséum National d’Histoire Naturelle of Paris. A search on the MNHN online database, so far comprising only the specimens kept in the Laboratoire but not those in the Zoothèque, indicated 51 specimens of the various subspecies *Pterocles exustus*. The oldest specimens are from Adolphe Boucard (1839–1903), therefore far too late to have been at Temminck’s disposal. No type specimen is believed to be present in the Zoothèque (Claire Voisin in litt. 16 November 2015). It is unknown whether the Verreaux’ specimen ZMB 11419 nowadays housed in Berlin is the specimen that Temminck saw in Paris.

## The problem

Distributions of *ellioti*, *olivascens*, *erlangeri* and *hindustan* are well established and do not present any nomenclatural or taxonomical issues in respect to the nominate subspecies. However, the situation between *floweri* and *exustus* is problematic and necessitates a review. When describing his *floweri*, from Upper Egypt and Fayum, Nicoll (1921) originally named it *Pterocles senegalensis floweri*, well aware that Lichtenstein’s *senegalensis* (Lichtenstein 1823a) ranged from Senegambia to Nubia (actual south Egypt and north Sudan). At the end of the 19^th^ century, Ogilvie-Grant (1893: 12) used already the name *exustus* Temminck instead of *senegalensis* Lichtenstein but the author did not give any reason. It was only a decade after Nicoll’s description that *Pterocles senegalensis* M.H.C. Lichtenstein, 1823, was found to be pre-occupied by *Tetrao senegalensis* Shaw, 1810 (Bannerman 1931: 290 footnote 1, see Dickinson et al. 2006: 343 for use of the date 1810 as well as the authorship), a junior synonym of *Tetrao senegallus* Linnaeus, 1771, and subsequently replaced by *Pterocles exustus* Temminck, 1825 (see e.g. Hartert 1912-1921: 1510, Bannerman 1931: 290, Hutson and Bannerman 1931, Mackworth-Praed and Grant 1937, Peters 1937: 4). Hartert (1912-1921: 1510), Mackworth-Praed and Grant (1937) and Peters (1937: 4) restricted the range of nominate *exustus* to Senegal as follows: “Senegal, Ägypten, Nubien. Terra typica Senegal”, “Senegal” and “West coast of Africa, Egypt and Nubia, = Senegal”, respectively. More recently, in regards to *floweri* distribution, Gouraud (2015) pointed out that the type locality of *exustus* should be clarified and recommended lectotypification of a non-Egyptian specimen from the *Pterocles exustus exustus* series (Arts. 73.2.3 and 76.2 of the Code).

### Lectotypification of *Pterocles exustus* Temminck, 1825

From the above discussion, the lectotype should be designated amongst specimens RMNH.AVES.87615, RMNH.AVES.87616, NMW 562, NMW 563, ZMB 11417 and ZMB 11418. By designating a lectotype, the Code (amended Art. 74 [see Declaration 44], Recommendation 74B) recommends that “other things being equal, an author… should give preference to a syntype of which an illustration has been published.” Temminck (in Temminck and Laugier de Chartrouse 1820: footnote, pl. 3; see Dickinson 2001: 46 for use of the date 1820) stated that “...to avoid useless repetition, specimens used for the plates are always housed in the first collection mentioned” [translated from French]. Thus, Temminck indubitably used Leiden specimens for the plates. We do not see any particular reason why those specimens should be the ones exchanged with NMW and ZMB. Therefore, specimens nowadays housed at Naturalis should be those used for plates 354 (male, AVES.87615) and 360 (female, AVES.87616). The first to have been published should be designated as lectotype. We do this here:

*Pterocles exustus* Temminck in Temminck and Laugier de Chartrouse 1825: pl. 354 and 360 + text.

**Figure 1.**
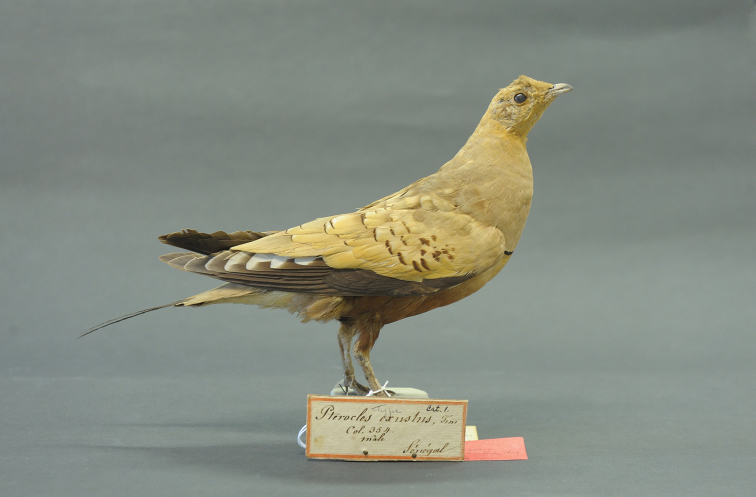
*Pterocles exustus* Temminck, 1825. Lectotype RMNH.AVES.87615. Photograph courtesy of Naturalis Biodiversity Center, Leiden, the Netherlands.

**Lectotype** (hereby designated): RMNH.AVES.87615 (Figure 1), adult male (mount), Senegal **(type locality)**. Collector unknown.

**Paralectotype**: RMNH.AVES.87616, adult female (mount), Senegal. Collector unknown.

**Paralectotype**: NMW 562, adult male (relaxed mount), Senegal. Collector unknown.

**Paralectotype**: NMW 563, adult female (relaxed mount), Senegal. Collector unknown.

**Paralectotype**: ZMB 11417, adult male (skin), Dongola, Sudan, collected at an unknown date: [= January-June 1822]. Collected by/for Hemprich and Ehrenberg.

**Paralectotype**: ZMB 11418, adult female (skin), Dongola, Sudan, collected at an unknown date: [= January-June 1822]. Collected by/for Hemprich and Ehrenberg.

Probable paralectotype: MLC.2011.0.1184, adult female (mount), Upper Egypt, Egypt. Collected by/for Rüppell.
